# Analysis of bamboo fibres and their associated dye on a freshwater fish host-parasite system

**DOI:** 10.1007/s11356-024-34626-7

**Published:** 2024-08-14

**Authors:** Scott MacAulay, Numair Masud, Jo Cable

**Affiliations:** School of Biosciences, Cardiff University, Cardiff, CF10 3AX UK

**Keywords:** Anthropogenic fibres, Fibre pollution, Alternative microplastics, Fish health, Host-parasite interactions

## Abstract

With the growth of the fashion and textile industries into the twenty-first century, associated pollution has become pervasive. Fibre-based microplastics are the most common types of plastics recovered from aquatic ecosystems encouraging the move towards organic fibre usage. Often marketed as biodegradable and ‘environmentally friendly’, organic textile fibres are seen as less harmful, but their impacts are understudied. Here, we assess the health effects of reconstituted bamboo-viscose fibres, processed bamboo-elastane fibres (both at 700 fibres/L) and their associated dye (Reactive Black-5, at 1 mg/L) on fish, with an emphasis on disease resistance utilising an established host-parasite system: the freshwater guppy host (*Poecilia reticulata*) and *Gyrodactylus turnbulli* (monogenean ectoparasite). Following 3 weeks exposure to the bamboo fibres and associated dye, half the experimental fish were infected with *G. turnbulli*, after which individual parasite trajectories were monitored for a further 17 days. Overall, exposures to reconstituted bamboo-viscose fibres, processed bamboo-elastane fibres or dye were not associated with any change in host mortality nor any significant changes in parasite infection burdens. When analysing the routine metabolic rate (RMR) of fish, uninfected fish had, on average, significantly impacted RMR when exposed to processed bamboo-elastane (increased RMR) and reconstituted bamboo-viscose (decreased RMR). Hosts exposed to reconstituted bamboo-viscose and the associated dye treatment showed significant changes in RMR pre- and post-infection. This study bolsters the growing and needed assessment of the potential environmental impacts of alternative non-plastic fibres; nevertheless, more research is needed in this field to prevent potential greenwashing.

## Introduction

The fashion and textile industries contribute significantly to environmental pollution via wastewater containing additives and other associated chemicals, in addition to fibres shed from clothing. This presents potential health concerns for humans, other animals and their environments due to direct or indirect exposure to textile waste (Kishor et al. 2021). Around 35% of oceanic microplastic pollution is attributed to the fashion industry, mostly non-biodegradable (i.e. unable to break down into substrates usable for microbial aerobic or anaerobic metabolism), synthetic origin (i.e. produced via chemical synthesis) and petroleum-based polymers (i.e. polymers derived from hydrocarbons) such as nylon, spandex and polyester (Boucher and Friot 2017; Suaria et al. 2020). Global textile production is dominated by petroleum-based synthetic fibres (~ 60% of total production) compared to naturally derived (~ 30% of total production) and other fibre types (~ 10% of total production) (Carr 2017). Petroleum-based fibre usage has risen with the advent of ‘fast fashion’ that produces billions of clothing items per year (Niinimäki et al. 2020). Fast fashion garments are only worn on average ten times before throwaway, where they are sent to landfill more often than are recycled (TRAID 2018; Barnardos 2021). The affordability of these garments comes at an environmental cost (Niinimäki et al. 2020). Non-degradable polymers commonly used in these garments constantly release fibres, which end up in water bodies through run-off, wastewater and airborne pathways (Liu et al. 2021). Regular household fibre waste generation alone can reach worrying scales, with fibre effluent counts reaching in the millions per wash, not accounting for industrial scale generators (Xu et al. 2018), such as netting from fishing equipment and masks from medical waste (Sillanpää and Sainio 2017; De Falco et al. 2019). One mitigation strategy to reduce this waste and its harmful effects is the drive toward more plant-derived (i.e. nature-based) products, which in theory are degraded *in-natura* by microbes compared to petroleum-based fibres which resist breakdown (Pekhtasheva et al. 2011; Arshad et al. 2014; Resnick 2019). It is essential, however, to ensure that products marketed as ‘ecologically friendly’ are less damaging to the environment by empirical testing under controlled conditions. Indeed, the EU Directive 2019/904 highlighted the potential problem of transitioning to non-plastic polymers, such as bamboo or hemp, without sufficient knowledge of their environmental and biological impact (Hann et al. 2020).

The negative impacts of granular microplastics on organisms are increasingly well documented (Wright et al. 2013a, 2013b; de Sá et al. 2018; Ockenden et al. 2021), but data on fibre exposure is limited. Granular petroleum-based microplastic consumption in fish not only increased their parasite burden but also increased host mortality (Masud and Cable 2023), and similar effects were seen following exposure to petroleum-based microplastic polyester fibres (concentration ~ 700 fibres/L) (MacAulay et al. 2023). In contrast, exposure to bamboo fibres (for 52 days) from a commercially available t-shirt, interestingly, significantly reduced parasite burdens in adult fish compared with fish not exposed to any fibres (MacAulay et al. 2023). Such work supports the drive to utilise plastic alternatives, with bamboo being a prime contender as a biobased polymer, leaving a lower carbon footprint and requiring less water during culturing than other biofibres such as cotton (Afrin et al. 2009; Waite 2010; Ogunwusi 2013). A further consideration regarding transitioning to alternative fibres is that fibres shed from commercial textiles (no matter the origin) are very different to raw non-processed fibres (Yaseen and Scholz 2019). Although bamboo is entirely cellulose-based, the rigidity of the plant means it requires considerable processing before it is suitable for textile use; hence, bamboo cellulose is chemically regenerated to increase malleability (Kauffman 1993). The resulting bamboo-viscose is then combined with a petroleum-based polymer, such as elastane, to increase flexibility and allow it to function as a comfortable textile garment.

The assumption that biobased fibres (derived directly from a biological source) are inherently ‘better’ might be an example of ‘Greenwashing’ (conveying false or misleading information regarding a product’s potential environmental impact; see de Freitas Netto et al. (2020)). Natural fibres, for instance, share sorbing capabilities with petroleum-based fibres (Ladewig et al. 2015; Stanton et al. 2019), and all finished products contain additives. Fibres, particularly those from textiles, have been altered and treated to meet functional requirements of the end product, which involve chemical alteration such as bleaching or dying (Holkar et al. 2016; Yaseen and Scholz 2019). Reactive dyes, frequently used in textiles, easily (and strongly) bind to common fibre types, such as cotton and wool (Chavan 2011; Shang 2013). These reactive dyes hydrolyse with water even without an auxiliary compound, such as salt, to produce dyed fabrics, whereas other dyes may require additional compounds to ensure fastness (Gopalakrishnan et al. 2019). When fabrics enter the aquatic environment, these additives have a greater likelihood of leaching from fibres into the water column. This suggests that dyes may interact with aquatic organisms in two ways: through direct consumption of the dyed fibre or passive consumption of the dye-tainted water. Dyes, including reactive dyes such as the widely used Reactive Black-5, have known negative impacts on aquatic organisms including developmental defects and cell death observed in zebrafish embryos (Manimaran et al. 2018; Joshi and Pancharatna 2019). This highlights how each aspect of textile pollution, from whole fibres to additives, must be considered in any ecological assessment.

Traditional ecotoxicological assessments typically overlook host-parasite interactions focussing on either cellular toxicity or whole organism level effects, without considering parasitism as the norm within ecosystems (Poulin 1999; Marcogliese and Giamberini 2013). Host-parasite interactions can be severely impacted via pollutants, many of which are immunosuppressant, and this includes particulate pollution such as microfibres (Sures 2006, 2008; Buss et al. 2022). Given that parasites are the dominant biomass within all ecosystems, this has consequences for host life history traits, including increased stress biomarkers, inhibited feeding, reduced predator evasion and survival (Kuris et al. 2008; Lefèvre et al. 2009). With the plethora of pollutants in wastewater, freshwater fish are often some of the first organisms exposed to contaminants which can be detrimental to their welfare. The effects of microplastics, fibres and their additives on fish include transcriptional changes, inhibited feeding and growth, reduced disease resistance and reduced survival (Limonta et al. 2019; Pannetier et al. 2020; Masud and Cable 2023; MacAulay et al. 2023). Adult male guppies (*Poecilia reticulata*) exposed to fibres released directly from a commercial bamboo-viscose (with elastane) garment experienced reduced parasite burdens whilst the leachate from these fibres had no impact on the parasite itself (*Gyrodactylus turnbulli*; see MacAulay et al. (2023)). Building on this, here we assess the impacts of both the whole and individual components of bamboo textile fibres. We tested reconstituted bamboo-viscose fibres, and processed bamboo-viscose with elastane fibres (from a commercially available black t-shirt) alongside a reactive black dye (commercially used in the textile industry) on juvenile fish metabolism, disease resistance and mortality.

## Methods

### Host-parasite system

We utilised the established guppy-*Gyrodactylus turnbulli* model for this study, which allows us to non-destructively monitor parasite burdens over time for individual hosts using a parasite with rapid (24–48 h) reproduction (Bakke et al. 2007). Size-matched mixed ornamental juvenile guppies (*n* = 240 laboratory strain, established in November 1997) were maintained within 70-L aquaria at 24 ± 0.5 °C on a 12-h:12-h light/dark photoperiod (lights on 7 am and off at 7 pm) prior to the investigation. For experimental infection, we utilised the *Gt3* strain of *G. turnbulli*, isolated from a Nottingham aquarium pet store and cultured under laboratory conditions since establishment in November 1997 (King and Cable 2007). All fish prior to experimental infections were measured (mean standard length = 13.2 mm, SE = 0.15, SD = 1.16) and weighed on an electronic scale by mildly anesthetising individuals with 0.02% MS-222.

### Fibre and dye preparation

The black bamboo fabric (from BAM Bamboo) was of the same origin as used in MacAulay et al. (2023) and consisted of 95% bamboo-viscose and 5% elastane. Bamboo fabric was cut into 7.5 cm^2^ squares, then shred into 0.5–1.5 cm^2^ pieces using sterile scissors and immersed in 1 L of dechlorinated water and agitated to promote fibre shedding to simulate a washing cycle. The same volume of raw reconstituted bamboo-viscose fibres (regenerated cellulose from bamboo plants) was agitated in 1 L dechlorinated water. A drop of each fibre water was then viewed under a compound microscope at 40 × magnification, and the number of fibres counted on days 1, 3, 5 and 7 of soaking. This was repeated 10 times per fibre treatment to calculate the average number per 1 mL, which were then all diluted to 700 fibres/L, equivalent to levels found in some natural systems (Carr 2017; Velasco et al. 2022). The reactive black dye, obtained from Sigma-Aldrich (Merck product code 306452), is analogous to the setazol black SDN dye previously confirmed by BAM Bamboo to be used during manufacture of bamboo clothing products. Wastewater has been found to contain concentration of dye upwards of 10 mg/L (Munagapati et al. 2018; Jalali Sarvestani and Doroudi 2020); due to ethical considerations, a concentration of 1 mg/L was utilised here.

### Experimental design

The experiment was conducted in two batches and batch effect was accounted for during statistical analysis. Fish were separated into four treatment groups: (1) control (*n* = 60), (2) processed bamboo-viscose t-shirt with 5% elastane (*n* = 60), (3) raw reconstituted bamboo-viscose fibres (*n* = 60) and (4) reactive black dye (*n* = 60).

**Fig. 1:**
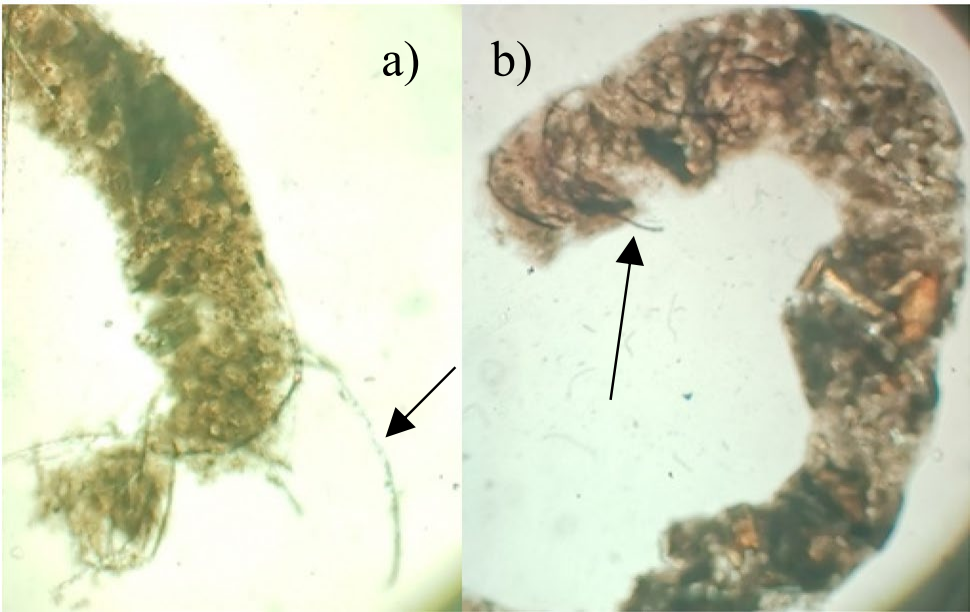
Faecal casings from fish (*Poecilia reticulata*) exposed to: **a**) raw bamboo-viscose fibres, where fibres are contained within and without the casing, and **b**) processed bamboo-elastane, with arrow indicating presence of individual fibres expulsed from the faecal casing after being compressed under a cover slip

A preliminary trial was conducted on *n* = 5 fish per fibre treatment where individual fish were isolated and maintained in 500-mL containers. Fish were exposed for 7 days to ~ 700 fibres/L for either reconstituted bamboo-viscose or processed bamboo-elastane fibres, equivalent to fibre loads found in some natural environments (Carr 2017). This involved adding 1 mL of the fibre mixture at the same time as adding ground-powdered food (Aquarian®) to each 500-mL container, both of which initially float but slowly sink as they absorb water. Control fish (*n* = 5) were maintained under the same conditions but without fibre exposure. Each day, faecal matter from the water was transferred using a glass pipette onto a pre-cleaned glass slide, crushed under a cover slip and observed under a dissecting microscope to confirm the presence of fibres encapsulated within the faeces (Fig. 1).

**Fig. 2:**
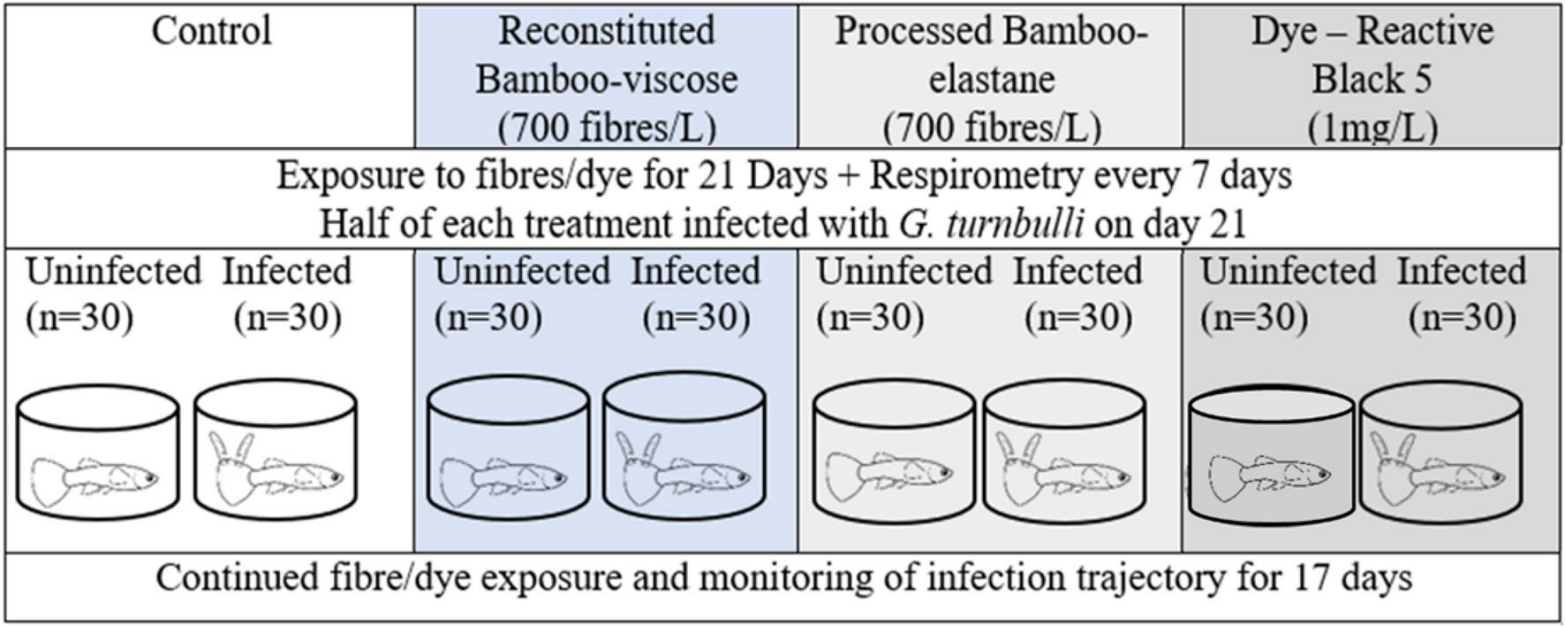
Schematic representation of experimental design. Four treatments: controls (exposed to only dechlorinated water), reconstituted bamboo-viscose (exposed to 700 fibres/L of reconstituted bamboo-viscose fibres), processed bamboo-elastane fibres (exposed to 700 fibres/L of bamboo-elastane fibres) and dye (reactive black 5 dye at 1 mg/L), where exposure was conducted for 21 days. On day 21, half the fish from each treatment were infected with two *Gyrodactylus turnbulli* and the infection trajectory monitored for a further 17 days whilst continuing previous treatment exposure

For the main experiment, all fish were isolated into 500-mL containers (i.e. 1 fish per 500-mL container) and exposed to fibres (i.e. ~ 700 fibres/L) or dye (concentration 1 mg/L) for 21 days (Fig. 2). Both fibre mixtures were agitated prior to exposure to ensure thorough mixing of the fibres within the water column for equal dispersion when introduced into the containers. Control fish were fed the same quantity of flake food (10% of body weight; Frederickson et al. 2021) without fibre or dye addition, to ensure that nutrition was not a confounding variable. Due to the exposure method (immersion), it is likely that consumption of fibres and dye occurred primarily passively, with active consumption probable but not verifiable (see Fig. 1). A full water change (for both the preliminary trial and main experiment) occurred every alternate day prior to feeding but after respirometry, which involved removing all water from the 500-mL containers in which fish were housed and replacing with fresh temperature controlled dechlorinated water. Upon refilling, the fish were then exposed to their respective treatment and fed the flake food (Aquarian®). During feeding, precaution was taken to ensure that the experimenters clothing did not contribute to fibre contamination by always wearing cotton short-sleeved clothing, but total elimination was not guaranteed (Gwinnett and Miller 2021).

### Experimental infection

After 21 days of fibre exposure, half of the fish in each treatment group were infected (*n* = 30 total) and half remained uninfected (*n* = 30 total). Fish to be infected with *G. turnbulli* were lightly anaesthetised with 0.02% MS-222 and then held in water alongside a donor fish. Using a dissecting microscope, with fibre optic lighting, two gyrodactylid worms were transposed to the caudal fin of the recipient fish following the standard methods of King and Cable (2007). Uninfected fish were anaesthetised and handled in the same manner without the introduction of parasites to control for any handling stress (sham infections). All infected and sham-infected fish were maintained within 500-mL containers throughout the experiment to ensure transmission was not a confounding variable for this experiment. Parasite numbers were assessed every 48 h for 17 days and this involved mildly anesthetising infected fish (using 0.02% MS-222) and counting the number of worms present under a dissecting microscope with fibre optic illumination (see King and Cable (2007) for detailed description). Fish were categorised as either Resistant (parasite numbers on a host fail to increase above 8 worms and most individual hosts cleared their infections), Responder (parasite numbers increased but then plateaued or decreased) or Susceptible (parasite numbers consistently increased) (see Bakke et al. (2002) for more in-depth explanation of these categories). The same feeding regimes continued during the infection phase of the experiment, i.e. both exposure treatments and foods. Any host mortalities were recorded throughout the study.

### Respirometry

To investigate whether exposure to either the fibres, dye or both impacted the routine metabolic rate (RMR) (Chabot et al. 2016), infected guppies (prior and during infection with *G. turnbulli*) (*n* = 24) were transferred to respirometer chambers on days 0, 7, 14, 21, 28 and 35 of exposure, with each treatment tested on the same exposure days but in batches of 4 fish. For day 21, when infections occur, respirometry was measured prior to infection. All measurements were conducted in a respirometry set-up that permitted monitoring of fish alongside a control simultaneously and temperature for the duration of measurements was maintained at 24 ± 0.5 °C. All water used for experimental purposes was autoclaved prior to use and then brought to the desired temperature. The static respirometry set-up consisted of individual glass chambers (130 mL, sealed DuranTM square glass bottles with polypropylene screw caps, Fisher), which were briefly washed with ethanol (Sigma-Aldrich) prior to commencing measurements to minimise background noise before the start of each respirometry trial. Chambers were fitted with individual contactless oxygen sensor spots attached to probes that were connected to a FireSting O_2_ meter (PyroScience, Aachen, Germany). The O_2_ concentration within respirometry chambers was measured every 1 s for 30 min total (10-min acclimation time and 20 min for recordings) using the following equation: $RMR= \frac{\Delta O2}{M} \times V\text{c}$, where *M* is fish mass in grams, *V*_c_ is the volume of the respirometer chamber in mL and ΔO_2_ is the rate of oxygen decline (Bonneaud et al. 2016) calculated as the slope of a linear regression. During respirometry, the O_2_ levels never dropped below 7 mg L^−1^ and were maintained within the recommended levels for freshwater tropical fish (OATA 2008). Each individual fish was weighed immediately following respirometry, but only prior to infection as weighing hosts with ectoparasites could influence parasite burdens. Following infections, the average weight increase (0.03 g for all treatments) was calculated and added onto the weights for measurements. All respirometry measurements were taken prior to any handling or water-changing stress.

### Ethics

All animal work was approved by the Cardiff University Animal Ethics Committee and conducted under UK Home Office licence PP8167141. All care was taken to minimise fish stress by implementing practices such as no netting, limiting noise, consistent light regime (i.e. 12-h:12-h light-darkness cycles) and water temperature (i.e. 24 °C) within temperature controlled, Home Office approved aquatic laboratories.

### Statistical analyses

All statistical analyses were carried out under RStudio version 4.2.3 (http://www.R-project.org/). For all statistical models described below, model assumptions were tested, specifically normality of standardised residuals and homogeneity of variance and all final models were chosen based on the lowest Akaike Information Criterion (http://CRAN.R-project.org/package=lme4).

#### Parasite metrics

For this study, the following response variables were measured in relation to parasite metrics: parasite count over time, maximum parasite burden, peak infection day, Area Under Curve (AUC), duration of infection and rate of parasite increase. Here, maximum parasite burden is defined as the maximum number of *G. turnbulli* worms at a particular time point, defined as peak infection day. To calculate AUC, a common pathogen metric utilised to quantify total pathogen burdens over the course of an entire infection trajectory, we utilised the trapezoid rule (White 2011). Rates of parasite increase, indicative of parasite reproduction, were calculated as the slope of the curve of individual infection trajectories. To analyse mean parasite intensity, maximum parasite burden, peak parasite day, AUC, average RMR and duration of infection, we utilised generalised linear models (GLMs). Standard length was initially included in the models, but as it did not explain significant variation it was removed from subsequent models, as part of model refinement (Thomas et al. 2013). For both mean parasite intensity and maximum parasite count, we used a GLM with a negative binomial error family and the log link function, within the *MASS* package (Venables and Ripley 2002) in R Studio. For analysing AUC sum, we had to transform the data using the Box-Cox transformation method also within the *MASS* package in R, as no family structure and link function could satisfy the assumptions of GLMs with the raw data, i.e. normality of standardised residuals and heterogeneity of variance. Subsequently, a GLM with a Gaussian error family and the identity link function was used, which did satisfy all model assumptions. A GLM with a Gaussian error family and the inverse root link function was used for analysing peak infection day. For the analysis of parasite count over time, where we needed to account for pseudo replication as the same fish was observed for parasite numbers over multiple time points, we utilised a generalised linear mixed model (GLMM) from the ‘lme4’ package (Bates et al. 2015). This was carried out as a negative binomial GLMM where treatment, day and the interaction day and treatment were our fixed factors and fish ID was included as the random factor.

#### Host metabolism

For analysing host metabolism, we assessed how mean routine metabolic rate (RMR) of fish varied between experimental treatments using a GLM with an inverse Gaussian family and the identity root link function. We analysed individual RMR trends using a GLMM with Gaussian family and identity link functions, where the treatment, day and the interaction between treatment and day were fixed factors and fish ID was included as a random factor. This GLMM was used to create a prediction plot using the *ggpredict* function within the ‘*ggeffects*’ package in R (Johnson and O’Hara 2014). In addition, emmeans post hoc analysis was applied to assess significance of day and treatment using the ‘*emmeans*’ package (Lenth and Lenth 2018).

## Results

### Host survival and disease burdens

**Fig. 3:**
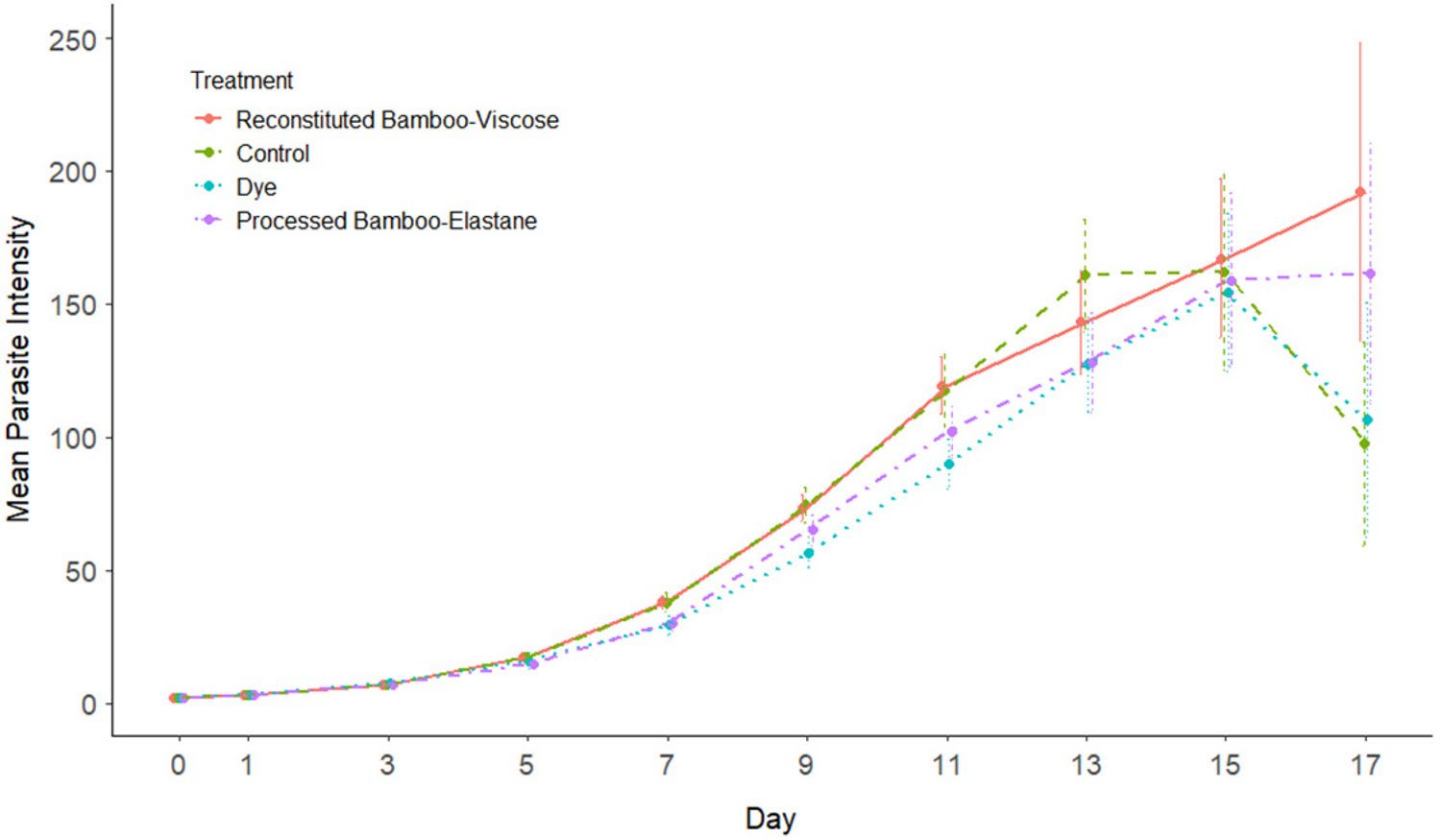
Mean parasite intensities of *Gyrodactylus turnbulli* per treatment (distinguished by colour and line type) per day (including standard error) on their host *Poecilia reticulata*

Neither reconstituted bamboo-viscose fibres, processed bamboo-elastane fibres nor RB5 dye had any significant impact on juvenile fish mortality, infected nor uninfected (GLM: *p* > 0.05), as number of deaths across treatments did not vary significantly. After 17 days of infection, there was no significant difference between the AUC sum, maximum parasite burden nor peak day between any of the treatments (*p* > 0.05) (Fig. 3). Treatment had no impact on the day in which the parasites reached their peak; however, Batch 2 reached peak day significantly earlier (approximately 3 days) than Batch 1 (GLM: Batch2, Est =  − 0.017, SE = 0.049, *p* = 0.0007). Parasite count over time was not significant between treatments (GLMM: reconstituted bamboo-viscose; SE = 10.858885, *t* = 0.2703594, *p* = 0.786883, dye; SE = 10.836791, *t* =  − 0.8526725, *p* = 0.3938409, processed bamboo-elastane; SE = 10.869252, *t* =  − 0.5640176, *p* = 0.5727422) nor was the interaction between day and treatment (GLMM: *p* > 0.05) whilst day was significant (GLMM: F_9, 240_ = 1573.9, *p* < 0.001). Infection status (Resistant, Responder or Susceptible) did not vary significantly between treatments (*Χ*^2^ = 7.1238, df = 6, *p* = 0.3095). In all treatments, the dominant status was that of Susceptible (control *n* = 17, raw bamboo-viscose *n* = 20, dye *n* = 19 and processed bamboo-elastane *n* = 22), followed by Responders (control n = 11, raw bamboo-viscose *n* = 10, dye *n* = 7 and processed bamboo-elastane *n* = 7), with the fewest (or none) being Resistant (control *n* = 2, raw bamboo-viscose *n* = 0, dye *n* = 4 and processed bamboo-elastane *n* = 1).

### Respirometry

**Fig. 4:**
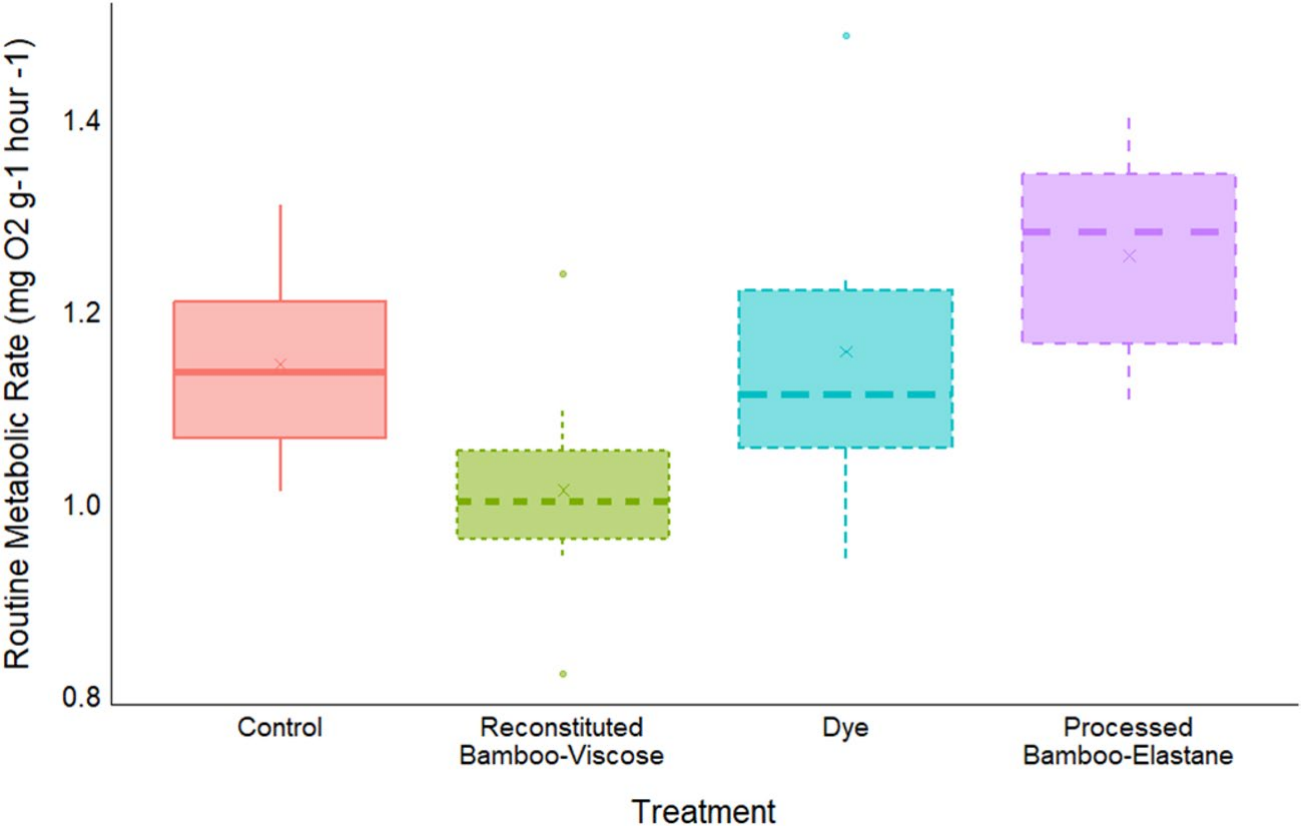
The average routine metabolic rate (RMR) of fish per treatment, accounting for all measurements taken for each treatment across the duration of the experiment. Box plot shows the median (line), mean (cross) interquartile range (box) and the 1.5 × interquartile range (whiskers). The filled circles represent values out with the 1.5 × interquartile range. Each box is outlined with different line types to represent each treatment

**Fig. 5:**
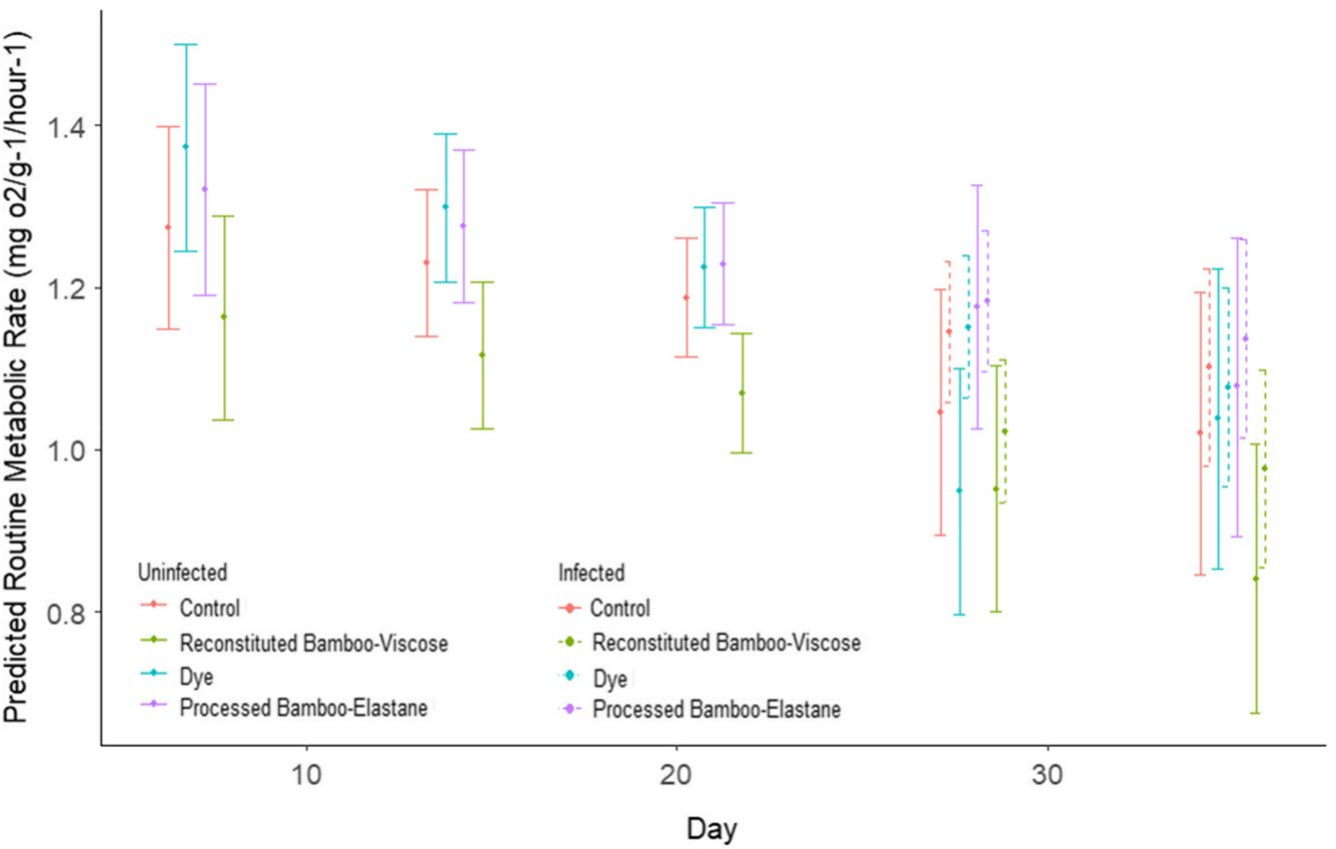
Predicted range of routine metabolic rates (RMRs) (mg O_2_/g^−1^/h^−1^) for fish, per treatment per day, across 38 days. Fish were exposed to their respective treatments across the entire experiment, but days 28 and 35 represent RMRs where some fish were actively infected (post day 21) with *Gyrodactylus turnbulli* (dashed error bars) and some remained uninfected (solid error bars)

The average RMR for control fish (across the duration of the experiment; Fig. 4) was 1.136 mg O_2_ g^−1^ h^−1^ fish^−1^, whilst fish exposed to reconstituted bamboo-viscose fibres had an average RMR of 1.052 mg O_2_ g^−1^ h^−1^ fish^−1^, processed bamboo-elastane fibres of 1.350 mg O_2_ g^−1^ h^−1^ fish^−1^ and dye of 1.182 mg O_2_ g^−1^ h^−1^ fish^−1^. This translated to no significant difference in the average RMR between control and dye exposed fish (GLM: Est = 0.025263, SE = 0.054156, *p* = 0.64617); however, the average RMR of processed bamboo-elastane exposed fish was significantly higher than control fish (GLM: Est = 0.207028, SE = 0.061548, *p* = 0.00326) whilst reconstituted bamboo-viscose exposed fish had significantly lower average RMR than control fish (GLM: Est =  − 0.111341, SE = 0.049450, *p* = 0.03638) (Fig. 4). For RMR, day 21 represents the final day of exposure without infection, and measurements for days 28 and 35 represent RMR during infection with *G. turnbulli*. The interaction between treatment and day was significant for RMR for all fish (GLMM; *p* < 0.05). Looking at within treatment differences when fish had been infected after 21 days of bamboo and dye exposure, there was no significant difference between control and processed bamboo-elastane RMRs across the experiment. However, for reconstituted bamboo-viscose, there were significant differences in their RMR between days 7 and 28 (emmeans: Est = 0.2943, SE = 0.106, *p* = 0.0443), and days 7 and 35 (emmeans: Est = 0.4040, SE = 0.111, *p* = 0.0030), and for the dye between days 7 and 14 (emmeans: Est = 0.3771, SE = 0.107, *p* = 0.0043), days 7 and 28 (emmeans: Est = 0.5471, SE = 0.107, *p* < 0.0001) and days 7 and 35 (emmeans: Est = 0.4577, SE = 0.120, *p* = 0.0015). These results indicate a treatment specific influence of infection on RMR for the reconstituted bamboo-viscose and dye treatments, but not for controls or processed bamboo-elastane (Fig. 5).

## Discussion

Textile pollutants, which include microfibres and their associated dyes, are pervasive within freshwaters, and understanding their biological impacts on freshwater organisms is important for any welfare assessment. Despite the prevalence of studies on non-degradable fibres, the dominant proportion of aquatic microfibres are cellulose-based and likely of anthropogenic origin, where it has been suggested that coloured cellulose-based textile fibres have been misidentified as microplastics for many years (Wesch et al. 2016; Cesa et al. 2017; Stanton et al. 2019; Suaria et al. 2020). This highlights the need to understand the potential impacts of cellulose-based textile fibres on aquatic environments. The current study suggests that reconstituted bamboo fibres, processed bamboo fibres and the raw dye associated with these fibres do not negatively impact disease susceptibility or host survival, at least following 38 days exposure. However, physiological impacts of fibre and dye exposure revealed that processed bamboo-elastane did impact metabolism by significantly increasing routine metabolic rate (RMR) compared to baseline control fish. Conversely, fish exposed to reconstituted bamboo-viscose showed a significantly lowered RMR, indicating that fibres and their associated dyes can impose metabolic stress on fish (e.g. Parker et al. 2021; Parker et al. 2023. We also reveal that infections had treatment-specific impacts on RMR, specifically for fish exposed to reconstituted bamboo-viscose and the Reactive Black-5 dye.

As bamboo is entirely comprised of cellulose, it is biodegradable, but it is extremely rigid in its base structure. As such, for textile usage, the base structure of bamboo has to be chemically regenerated, and this reconstituted bamboo-viscose is considered semi-natural or semi-synthetic (Bien 2021). Elastane is then added to provide flexibility to the fabric (Kauffman 1993). Previously, we demonstrated that exposure of adult male guppies for 52 days (21 days prior to infection plus 31 days exposure during infection) to processed bamboo-elastane fibres resulted in lower *G. turnbulli* burdens, along with no adverse impact on mortality of the guppy host nor the parasite (MacAulay et al., in press). The current study time focussed on juvenile fish exposed for a shorter (38 days) period (21 days prior to infection plus 17 days during infection). From our current results, we reveal no impact of either reconstituted bamboo-viscose nor the processed bamboo fibres on disease dynamics using the same host-parasite system; however, we add to this knowledge by revealing that changes in metabolism are detectable when fish are exposed to processed bamboo-elastane and reconstituted bamboo-viscose in the same host-parasite system. Juvenile guppies exposed to microplastics for 28 days retain particles within the gut (Huang et al. 2020), which likely also occurs for fibres (in future, this could be confirmed through microscopy of the intestine), indicating the importance of assessing long-term impacts. Exposure to microplastics can stimulate juvenile fish immunity, potentially priming their immune system for infection (Huang et al. 2020). If this was the case in the current study, this might explain why these juveniles tolerated *G. turnbulli* infection more effectively than adult fish. The key difference between this and the previous study (MacAulay et al. 2023) is the life stage of the fish, which we know can influence immune response, where juvenile fish have a generally underdeveloped immune system versus the established immune system of mature fish (Zapata et al. 2006; Uribe et al. 2011). Despite no observable impacts being found in the current study, micro- and nano-level effects were not directly assessed: techniques such as histopathology, ELISA and transcriptomics may reveal impacts at the organ, cellular and DNA level (Petitjean et al. 2019; Huang et al. 2020).

The dye utilised here was Reactive Black-5 (RB5; also known as Remazol Black B), a readily available black dye commonly used in textile colouring. The wet fastness of reactive dyes is touted as a benefit, but when fibres dyed with these reactive dyes enter the water column (be that during washing or as waste products), it is possible to visually observe dye leaching out of the fibres and into the water (MacAulay et al. 2023). The dyes, and any other associated chemicals contained within the textile, will leach out of the fibres and enter the water column as leachate. In zebrafish, textile leachate and RB5 specifically can induce cytotoxicity within cell lines, cause malformations during larval development and increase mortality of embryonic fish (de Oliveira et al. 2016; Manimaran et al. 2018). These dyes can also impact behaviour, reducing activity in rainbow trout and competitive behaviours in fathead minnows following exposure to wastewater effluent *in-natura* (Garcia-Reyero et al. 2011; Almroth et al. 2021). Dye concentrations have been found to cause observable effects, under laboratory conditions, at concentrations greater than 1 mg/L, although 1 mg/L did increase mortality of zebrafish embryos (Manimaran et al. 2018). Within our study, fish parasite burdens were not impacted following exposure to 1 mg/L dye and no other observable detrimental effects were observed; however, this may be limited by the experimental duration and further investigations over a longer exposure period may be necessary. Malformations observed in previous studies focussed on larval zebrafish arguably at greater risk of developmental difficulties (Kato et al. 2004; Rojo-Cebreros et al. 2018) than the juvenile fish used within our study. RB5 is degradable by bacteria, which break down and decolour the dye within water (El Bouraie and Din 2016). The aquarium water used for the current study was not sterile but as it was completely changed and re-dosed every 2 days so it is unlikely that bacterial breakdown would have deactivated the dye during our experiment, but we acknowledge some breakdown products may have been generated.

For average routine metabolic rate (RMR), we observed no significant differences between dye-exposed fish and controls, but we did observe differences for reconstituted bamboo-viscose and processed bamboo-elastane-exposed fish. We recorded a lower average RMR for the reconstituted bamboo-viscose-exposed fish and a higher average RMR for processed bamboo-elastane exposed fish, compared to control fish. This suggests that reconstituted bamboo-viscose is associated with metabolic depression, although the reason for this is unclear. We know that freshwater fish often have cellulose-based detritus in their diets and that they can digest and utilise the nutrients from bamboo (Magurran 2005; Saha et al. 2006), which may explain why fish exposed to processed bamboo-elastane had significantly increased metabolism. It is plausible that the processed bamboo-elastane fibres, which are associated with multiple additives, including RB5 (which we tested in this study), were causing metabolic stress, seen here as a significant increase in RMR. Processed fibres, such as our processed bamboo-elastane, often contain chemicals such as sodium hydroxide, formaldehyde and hydrogen peroxide (Yaseen and Scholz 2019), all of which may influence RMR (Tavares-Dias 2021; Wood et al. 2021) but we are unable to say for certain. Here, we show that parasitic infections have a treatment-specific influence on RMR, where the RMR is depressed, matching the trend seen for other fish with parasitic infections (Hvas et al. 2017; Guitard et al. 2022; Schaal et al. 2022). In terms of temporal RMR variation across the experiment, we did not see a difference in RMR for fish between pre- (days 0–21) and post-infection (days 28 and 35) that were not exposed to fibres nor dye, whereas previous results indicate an increase in RMR with infection of *G. turnbulli* (see Masud et al. (2020); Robison-Smith et al. (2024)). This could be due to two factors: fish strain variation and life stage. The RMR of wild-origin fish under similar lab conditions was lower on average than the ornamental strain used here, and the wild-origin fish showed increased oxygen consumption post-infection (Masud et al. 2022; Robison-Smith et al. 2024). We used juvenile fish, which may have been under increased energy demands needed for maturation and sexual development, and as such infection did not significantly influence the RMR (Jobling 1994; Pichavant et al. 2001). Smaller fish will display a higher metabolic rate than larger fish, when weight is considered (Urbina and Glover 2013; Guitard et al. 2022). Overall, the decreased trend observed in RMR across the experiment (Fig. 5) matches what we would expect and is due to a combination of infection and growth, which directly correlates to lower RMR, as the fish were growing, gaining ~ 0.003 g per week (Urbina and Glover 2013).

Our current knowledge of fibre pollution is lacking. Most studies pertaining to fibre pollution focus on assessing the type and scale of fibre pollution, typically within a ‘plastic focus’ framework (e.g. Collard et al. (2017); Halstead et al. (2018); Henry et al. (2019); Ross et al. (2021)). This work does, however, highlight the sheer pervasiveness of fibres (Collard et al. 2017; Pazos et al. 2017; Ragusa et al. 2021; Ross et al. 2021), supporting the need for continued and improved assessment of their functional impact. Whilst 60% of fibres produced are synthetic (Carr 2017), and as such can be classified under the microplastic umbrella, the notoriety of microplastics has driven an upsurge in ‘alternative’ or biobased fibre types to reduce plastic pollution and their negative impacts. This work assesses the potential for bamboo fibres as a ‘green alternative’ to plastic fibres, by testing their impacts on freshwater fish metabolism and disease resistance. The results of this study do not give an unadulterated green light for these fibres as they highlight that even nature-based fibres may be detrimental for fish welfare over extended periods of exposure.

## Data Availability

All data pertaining to this manuscript will be made available upon contact of the corresponding author.
